# Self-Selected or Mandated, Open Access Increases Citation Impact for Higher Quality Research

**DOI:** 10.1371/journal.pone.0013636

**Published:** 2010-10-18

**Authors:** Yassine Gargouri, Chawki Hajjem, Vincent Larivière, Yves Gingras, Les Carr, Tim Brody, Stevan Harnad

**Affiliations:** 1 Institut des Sciences Cognitives, Université du Québec à Montréal, Montréal, Québec, Canada; 2 Observatoire des Sciences et des Technologies, Université du Québec à Montréal, Montréal, Québec, Canada; 3 Canada Research Chair in the History and Sociology of Science, Université du Québec à Montréal, Montréal, Québec, Canada; 4 Canada Research Chair in Cognitive Sciences, Université du Québec à Montréal, Montréal, Québec, Canada; 5 School of Electronics and Computer Science, University of Southampton, Southampton, United Kingdom; Northeastern University, United States of America

## Abstract

**Background:**

Articles whose authors have supplemented subscription-based access to the publisher's version by self-archiving their own final draft to make it accessible free for all on the web (“Open Access”, OA) are cited significantly more than articles in the same journal and year that have not been made OA. Some have suggested that this “OA Advantage” may not be causal but just a self-selection bias, because authors preferentially make higher-quality articles OA. To test this we compared self-selective self-archiving with mandatory self-archiving for a sample of 27,197 articles published 2002–2006 in 1,984 journals.

**Methdology/Principal Findings:**

The OA Advantage proved just as high for both. Logistic regression analysis showed that the advantage is independent of other correlates of citations (article age; journal impact factor; number of co-authors, references or pages; field; article type; or country) and highest for the most highly cited articles. The OA Advantage is real, independent and causal, but skewed. Its size is indeed correlated with quality, just as citations themselves are (the top 20% of articles receive about 80% of all citations).

**Conclusions/Significance:**

The OA advantage is greater for the more citable articles, not because of a quality *bias* from authors self-selecting what to make OA, but because of a quality *advantage*, from users self-selecting what to use and cite, freed by OA from the constraints of selective accessibility to subscribers only. It is hoped that these findings will help motivate the adoption of OA self-archiving mandates by universities, research institutions and research funders.

## Introduction

The 25,000 peer-reviewed journals and refereed conference proceedings that exist today publish about 2.5 million articles per year, across all disciplines, languages and nations. No university or research institution anywhere, not even the richest, can afford to subscribe to all or most of the journals that its researchers may need to use [1]. As a consequence, all articles are currently losing some portion of their potential research impact (usage and citations), because they are not accessible online to all their potential users [2].

This is supported by recent evidence, independently confirmed by many studies, to the effect that articles whose authors have supplemented subscription-based access to the publisher's version by self-archiving their own final draft to make it accessible free for all on the web (“Open Access”, OA) are cited significantly more than articles in the same journal and year that have not been made OA. This “OA Impact Advantage” has been found in all fields analyzed so far – physical, technological, biological and social sciences, and humanities [3]–[12]

Hence OA is not just about public access rights or the general dissemination of knowledge: It is about increasing the impact and thereby the progress of research itself. A work's research impact is an indication of how much it contributes to further research by other scientists and scholars – how much it is used, applied and built upon [13]–[17]. That is also why impact is valued, measured and rewarded in researcher performance assessement as well as in research funding [18].

### Self-archiving mandates

Only about 15–20% of the 2.5 million articles published annually worldwide are being self-archived by their authors today [8], [19]. Creating an Institutional Repository (IR) and encouraging faculty to self-archive their articles therein is a good first step, but that is not sufficient to raise the self-archiving rate appreciably above its current spontaneous self-selective baseline of 15–20% [20]. Nor are mere requests or recommendations by researchers' institutions or funders, encouraging them to self-archive, enough to raise this 20% figure appreciably, even when coupled with offers of help, rewards, incentives and even offers to do the deposit on the author's behalf [21]. In two international, multidisciplinary surveys, 95% of researchers reported that they would self-archive if (but only if) *required* to do so by their institutions or funders. (Eighty-one percent reported that, if it was required, they would deposit *willingly*; 14% said they would deposit reluctantly, and only 5% would not comply with the deposit requirement; [22].) Subsequent studies on actual mandate compliance have gone on to confirm that researchers do indeed do as they reported they would do, with mandated IRs generating deposit rates several times greater than the 20% self-selective baseline and well on the road toward 100% within about two years of adoption [20].

Universities' own IRs are the natural locus for the direct deposit of their own research output: Universities (and research institutions) are the universal providers of all research output, in all scientific and scholarly disciplines; they accordingly have a direct interest in hosting, archiving, monitoring, measuring, managing, evaluating, and showcasing their own research output in their own IRs, as well as in maximizing its uptake, usage, and impact [23], [24]. OA self-archiving mandates hence add visibility and value at both the individual and institutional level [25].

In 2002, The University of Southampton's School of Electronics & Computer Science (ECS) became the first in the world to adopt an official self-archiving mandate. Since then, a growing number of departments, faculties and institutions worldwide (including Harvard, Stanford, and MIT) as well as research funders (including all seven UK Research Funding Councils, the US National Institutes of Health, and the European Research Council) have likewise adopted OA self-archiving mandates. Over 160 mandates had already been adopted and registered and charted in the Registry of Open Access Repository Material Archiving Policies (ROARMAP) as of summer 2010.

In 2008, mindful of the benefits of mandating OA, the council of the European Universities Association (EUA, consisting of more than 800 universities, in 46 countries) unanimously recommended that all European Universities should create IRs and should require all their research output to be deposited in them immediately upon publication (to be made OA as soon as possible thereafter). The EUA further recommended that these self-archiving mandates be extended to all research results arising from EU research project funding. A similar recommendation was made by EURAB (European Research Advisory Board). In the US, the FRPAA has proposed similar mandates for all research funded by the major US research funding agencies.

Some studies, however, have suggested that the “OA Advantage” might just be a self-selection bias rather than a causal factor, with authors selectively tending to make higher-quality (hence more citable) articles OA [26]–[29]. The present study was carried out to test this hypothesis by comparing self-selected OA with mandated OA on the basis of the research article output of the four institutions with the longest-standing OA mandates: (i) Southampton University (School of Electronics & Computer Science) in the UK (since 2002); (ii) CERN (European Organization for Nuclear Research) in Switzerland (since November, 2003); (iii) Queensland University of Technology in Australia (since February 2004); (iv) Minho University in Portugal (since December, 2004).

## Methods

The objective was to compare citation counts – always within the same journal/year – for OA (O) and non-OA (Ø) articles, comparing the O/Ø citation ratios for OA that had been self-selected (S) vs. mandated (M). (The critical comparisons of interest were hence SO/Ø vs. MO/Ø.) The sample covered articles published between 2002 and 2006. The metadata for the articles were collected from the four institutional repositories, as well as from the Thomson-Reuters citation database. (Citation counts were extracted from the Thomson-Reuters database November, 2008. About two years need to elapse for the citations from the most recent year to stabilize.)

The effect of OA on citation impact cannot be reliably tested by comparing OA and non-OA journals because no two journals have identical subject matter, track-records and quality-standards (nor are there as yet enough established OA journals in most fields). The comparison must hence be between OA and non-OA articles published within the same (non-OA) journals [5]. For each of the mandated articles, M_i_, deposited in our four mandated IRs, we accordingly collected, as our pool of nonmandated controls for comparison, the N_j_ articles that had been published in the same journal, volume and year. Our sample of self-archived articles from 2002 to 2006 was distributed across 1,984 non-OA journals in the Thomson-Reuters database (**Table 1**). (Based on the Directory of Open Access Journals (DOAJ), 2% of journals indexed by Thomson-Reuters in 2006 were OA journals. All articles from these journals were removed from our pool because for them O/Ø comparisons were not possible.)

**Table 1:** Journal counts per year.

	Journal Count
**2002**	331
**2003**	367
**2004**	415
**2005**	445
**2006**	426
**TOTAL**	1,984

Number of journals in our sample for each year tested.

To reduce our nonmandated comparison sample to a reasonable processing size, we restricted the number of journal/year-matched controls to the 10 Ø_j_ articles that were semantically closest to their corresponding target M_i_ (as computed on the basis of shared words in their titles, omitting stop words). This tightening of content similarity also made the control articles even more comparable to their targets than using the full spectrum of same-journal content. The total size of the article sample (6215 mandated targets plus their 20,982 corresponding controls) from 2002 to 2006 was 27,197. (When more than one M article was published in the same journal/volume/year (which represents 66% of M articles), only 10 articles were selected as controls, using keyword matching for one of these M articles.)

**Figure 1:**
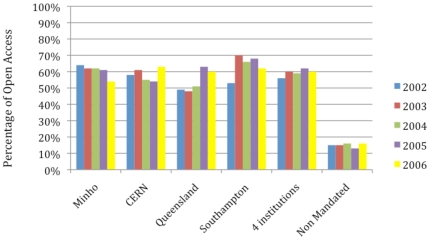
Open Access (OA) Self-Archiving Percentages for Institutions With Self-Archiving Mandates Compared to Non-Mandated, Self-Selected Controls. As estimated from the portion of their yearly published article output that is indexed by Thomson-Reuters, in this 2006 sample at least 60% of each of the four mandated institutions' total yearly article output was self-archived and hence made OA, as mandated. The corresponding percentage OA among the control articles published in the same journal/year (but originating from other, presumably nonmandated institutions) was 15%, or close to the frequently reported global spontaneous baseline rate of about 15–20% for self-selected (nonmandated) self-archiving [19]. In other words, about 15% of these papers were self-selectively self-archived when it was not mandated, whereas at least 60% were self-archived when it was mandated.

The full-text OA status of the articles in our sample was verified using an automated webwide search-robot [8] as well as an automated Google Scholar search. (Note that any OA articles that our robot missed would reduce any OA Advantage. Hence our estimate of the OA Advantage is conservative.) **Figure 1** shows each of our four mandated institutions' verified annual OA article deposits as a percentage of the institution's total published article output for each year based (only) on those articles published in the journals indexed by the Thomson-Reuters citation database; the resulting estimate of the overall OA mandate compliance rate is about 60%.(for publishing years 2002–2006, with the deposits up to 2009, when the analysis was conducted). Note also the robot data's confirmation of the approximately 15% baseline for spontaneous, self-selected (i.e., non-mandated) OA self-archiving among the control articles in the same journal/years [19].

This mandated deposit rate of 60% is substantially higher than the self-selected deposit rate of 15–20%. Of course, with anything short of 100% compliance it remains a logical possibility to hold onto the hypothesis that the OA citation advantage could be solely a self-selection bias by arguing that, when self-archiving is mandated, what used to be a bias toward self-selectively self-archiving one's *more* citable articles instead takes the form of a selective bias toward noncompliance with the mandate for one's *less* citable articles. But in that case a reasonable expectation would be at least a substantial reduction in the size of the OA impact advantage with a mandated self-archiving rate three times as high as the spontaneous self-archiving rate, were it indeed true that the OA advantage was solely or largely due to self-selection bias.

To test whether mandated OA reduces the OA citation advantage, 4 kinds of articles need to be compared:

- O M: OA, Mandated,
- Ø M: Non-OA, Mandated,
- O S: OA, Self-Selected
- Ø S: Non-OA, Self-Selected

The analysis uses the citation counts within each journal/year. Because the date on which the mandate was first adopted varies (from 2002 to 2004) for the four institutions, we analyzed the data for the four institutions jointly as well as individually. The individual analyses show the time-course of mandate compliance more clearly; the global analysis combines data, enlarges the sample size and smoothes out incidental effects of institutional and timing differences.

We compared the following ratios: O/Ø, OM/OS, OS/ØS, OM/ØM, OM/Ø, OS/Ø and OM/OS using their mean log citation ratios. For example, to compare mandated OA with self-selected OA, we computed the logarithm of the ratio OM_j_/OS_j_ for each journal *j* and then we computed the arithmetic mean of all the logarithms of those ratios for all journals. With OA/OS, there would be an advantage in favor of OM if the logarithm of the ratio was greater than zero, and in favor of OS otherwise.
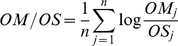
The logarithm is used to normalize the data and to reduce any effect arising from articles that have relatively high citation counts, compared to the whole sample. The comparisons are all within-journal, to minimize between-journal differences in content, quality and average citation levels (“journal impact factor”); OA articles are keyword-matched to their non-OA controls in order to minimize any differences still further.

## Results

**Figure 2:**
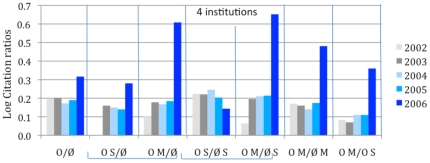
Log Citation Ratios Comparing the Yearly OA Impact Advantage for Self-Selected vs Mandatory OA 2002–2006. *O = OA article (Open Access)*; Ø = non-OA article (non-Open Access); M = Mandated OA; S = Self-Selected OA. Averages across the sample of four institutions with self-archiving mandates confirm the significantly higher citation counts for OA articles (symbolized here as “O”) compared to matched control non-OA articles (symbolized here as “Ø”) published in the same journal and year. They are compared as O/Ø log ratios in the seven comparisons. (The first comparison, O/Ø, for example, is the arithmetic mean of all the (log) ratios O/Ø for each of the 5 years.) OA articles are more highly cited irrespective of whether the OA is Self-Selected (S) or Mandated (M). The O/Ø Advantage is present for mandated OA (OM/ØS) and is of about the same magnitude irrespective of whether we compare the S ratios with the M ratios for the entire control sample (OS/Ø vs OM/Ø) or just compare S alone with M alone (OS/ØS vs OM/ØM). (The larger values for year 2006 are almost certainly due to the fact that 2006 was still too near to have stabilized at the time this analysis was conducted (2008–9); the analysis has since been extended for years 2006–2008, thereby stabilizing the data for 2006 and 2007, and yields the same results, always with the exception of the most recent year, which was 2008 in the most recent analysis.)

Overall, OA articles are cited significantly more than non-OA articles, confirming the repeatedly observed OA Advantage (O/Ø). There is also no evidence at all that mandated OA (OM) has a smaller citation advantage than self-selected OA (OS). **Figure 2** shows the results for the four institutions together. **Appendix S1** shows each institution separately. The pattern for the individual institutional data is largely the same as for the average across the four institutions.

For all OA vs Non-OA (O/Ø) comparisons, regardless of whether the OA was Self-Selected (S) or Mandated (M), the mean log citation differences are significantly greater than zero (based on correlated-sample t-tests for within-journal differences; **Table 2**). There is no detectable reduction in the size of the OA Advantage for Mandated OA (60%) compared to Self-Selected OA (15%).It would require a very complicated argument indeed (“self-selective noncompliance for less citable articles”) to resurrect the hypothesis that the OA Advantage is only or mostly a self-selection bias in the face of these findings. (Such an argument does remain a logical possibility until there is 100% mandate compliance, but an increasingly implausible one.)

**Table 2:** Paired Samples Test.

		Paired Differences							
					95% Confidence Interval of the Difference				
		Mean	Std. Deviation	Std. Error Mean	Lower	Upper	t	df	Sig. (2-tailed)
Pair 1	O - Ø	1.282	7.402	0.250	0.791	1.772	5.125	875	0.000
Pair 2	OS - Ø	0.890	7.475	0.295	0.312	1.469	3.023	643	0.003
Pair 3	OM - Ø	0.705	6.871	0.286	0.145	1.266	2.470	578	0.014
Pair 4	OS - ØS	0.809	7.535	0.297	0.225	1.393	2.719	641	0.007
Pair 5	OM - ØS	0.647	6.921	0.289	0.080	1.214	2.242	574	0.025
Pair 6	OM - ØM	1.540	9.526	0.684	0.191	2.888	2.251	193	0.026
Pair 7	OM - OS	0.834	7.898	0.421	0.006	1.662	1.982	351	0.048

Significance levels for the 2-tailed t-tests for the 7 differences graphed as log ratios in **Figure 2**, averaged across 2004–2006 (mandates began to be adopted in 2004). Open Access (O) vs. Non-Open Access (Ø); Mandated (M) vs. Self-Selected (S). The OA Advantage occurs irrespective of whether the OA is Self-Selected or Mandated.

### Logistic regression

The number of citations an article receives can be correlated with and hence influenced by a variety of variables. Those variables, in turn, could create another kind of bias. For example, older articles tend to have more citations than younger articles simply because there has been more time to cite them. If OA articles tended to be older than non-OA articles, then article age, rather than OA, could be the cause of the OA Advantage. A way to test whether correlates of citation other than OA are responsible for the OA Advantage is to perform a multiple logistic regression analysis to see whether OA alone is still significantly correlated with higher citations when the correlation with other variables has been “factored out.”

In ordinary multiple regression analysis, there might be, say, three “Predictor” variables used to predict a 4th “Target” variable. For example, in weather forecasting, each of (P1) temperature, (P2) pressure, and (P3) humidity is individually correlated with, and hence predictive of (T) rain. These three pairwise correlations are each examples of simple regression. The prediction is much better, however, if we use all three predictors jointly. This is called multiple regression. It gives each of the predictors a “weight” (ß) that estimates how much it contributes independently to predicting rain, with the other 2 predictors factored out. Multiple regression analysis works if the variables are continuous (like temperature) and normally distributed (i.e., bell-curve-shaped). But if the variables are discrete or not normally distributed, a variant analysis called logistic regression is used in which the variables are subdivided above and below a cut-off point, and various different models, with different cut-off points, are tested to see which ones predict the target variable the best in each range. We use this variant analysis, because our variables are not all continuous or normally distributed. The logistic regression weights (Exp(ß)) are estimates of the size of the individual contributions of each of our predictor variables to our target variable (citations).

(In **Table 3** – and in all the other Tables displaying the Exp(ß) weights for our logistic regressions – the relative size of the Exp(ß) weight for each of our 15 predictor variables (in each of our models, which vary in their ranges and cut-off points) estimates how much (and in what direction) each predictor contributes to predicting the target (citations); statistically significant contributions are in boldface. To visualize the size and the direction of the independent contributions of our predictors, each Table has a corresponding Figure, showing the contributions as color-coded bars.)

**Table 3:** Set of fourteen variables (plus one interaction) potentially influencing citation counts.

	Variable	Description
1	Age	How old is the article (articles published from 2002 to 2006)?
2	JIF	What is the Thomson-Reuters “Impact Factor” (average citations per article in 2-year window) of the journal in which the article was published (from 0 to 30)?
3	Auth_N	How many co-authors does the article have?
4	Ref_N	How many references does the article cite?
5	Page_N	How many pages in the article?
6	Sci	Is the article classified by Thomson-Reuters as Science (1) or Social Science (0)
7	Review	Is the article classified by Thomson-Reuters as a “review” article (1) or not (0)?
8	USA	What is the country of the first author (USA 1, other 0)?
9	OA	Is the article Open Access (1) or Not (0)?
10	Age*OA	The interaction between Age and OA
11	M	Does the author's institution Mandate Open Access (1) or Not (0)?
12	CERN	Is the first author from CERN (1/0)?
13	South	Is the first author from Southampton (1/0)?
14	Minho	Is the first author from Minho (1/0)?
15	Queens	Is the first author from Queensland University of Technology (1/0)?

We have accordingly analyzed the following set of variables that are potentially influencing citations. Variables 1–8 are known to be correlated with citation counts. Variable 9 is OA itself; and variable 10 is a measure of the degree to which the relation between OA and Age is non-additive. Variable 11 indicates whether or not the OA is mandated. Variables 12–15 are just the four mandating institutions that are our reference points in this study.

**Figure 3:**
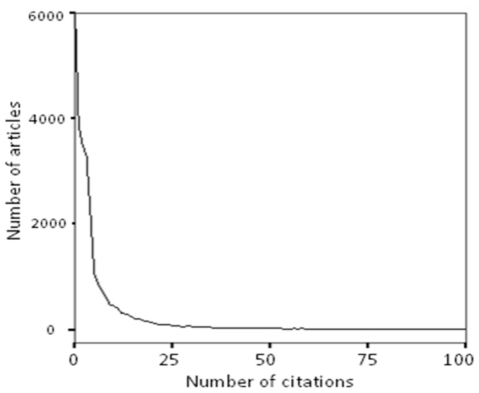
Distribution of citation counts (minus self-citations) for articles. Citation counts are not normally distributed. Of our sample of 27,197 articles, 23% had zero citations; 51% had 1–5 citations; 12% had 6–10 citations; 8% had 11–20 citations; and 6% had 20+ citations. It is for this reason that a logistic analysis rather than an ordinary regression analysis was conducted. (Cf. **Figure 4**, which presents the distribution of average Journal Impact Factors – which are, roughly, average citation counts – for journals.)

**Figure 4:**
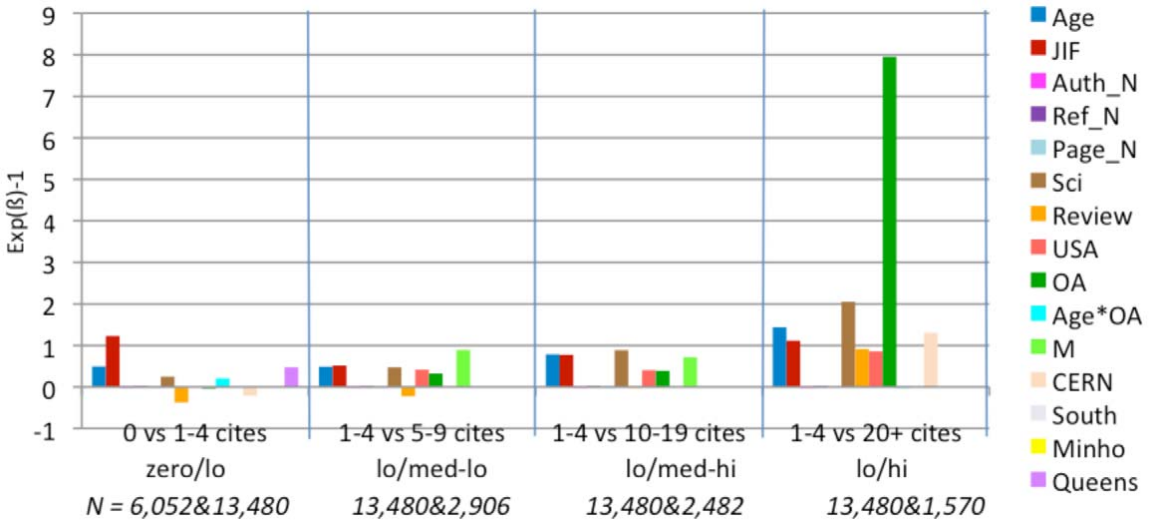
Exp(ß)-1 values for logistic regressions. These comparisons are based on 4 models, each analyzing a different comparison range. For each comparison (e.g., 1–4 citations (lo) vs. 5–9 citations (med-lo)) an article is assigned zero if its citation count is in the lower of the two ranges and one if it is in the upper range. Then the model assigns the best fitting weights to each of the fifteen predictor variables in their joint prediction of the citation counts. The weights are proportional to the independent contribution of each variable. (Only statistically significant weights are shown.) In most of the four citation range comparisons (zero/lo, lo/med-lo, low/med-hi, lo/hi), citation counts are positively correlated with Age, Journal Impact Factor, Number of Authors, Number of References, Number of Pages, Science, Review, USA Author, OA, and Mandatedness. There is also a significant OA*Age interaction in the top and bottom range. (Citations grow with time; for age-matched articles, the OA Advantage grows even faster with time; **Figure 6**). OA is a significant independent contributor in three of the four models and their citation ranges, especially in the the lo/hi comparison.

All self-citations were subtracted from the citation counts. (About 32% of the articles in our sample have at least 1 self-citation, with an average of about 2 self-citations per article.) As is well-known, and evident from **Figure 3**, citation counts are not normally distributed and instead follow a power-law or stretched-exponential function [19], [30], [31]. We accordingly used binary stepwise logistic regression analysis, with a dichotomous dependent variable, selecting for each test the model that maximizes the chi-square likelihood ratio. To make the interpretation of the coefficients easier, we exponentiated the ß coefficients (**Exp(ß)**) and interpreted them as odds-ratios (minus 1, to highlight the polarity of any change). For example, we can say for the second model (M2) that for a one unit increase in OA, the odds of receiving 5–9 citations (versus 1–4 citations) increased by +.323 (i.e., a factor of 1.323). **Table 4** and **Figure 4** show (Exp(ß)-1) values for each model with “**x–y cites vs. y–z cites**” as dependent variables ((x,y,x) ∈ {1, 2, 3, …, 20}), assigning 1 if the citation count (minus self-citations) was between y and z and 0 if it was between x and y. The four models comparing citation ranges are: (M1) zero vs. lo (1–4); (M2) lo (1–4) vs. med-lo (5–9); (M3) lo (1–4) vs. med-hi (10–19); (M4) lo (1–4) vs. hi (20+). (The Exp(ß) values of the variables turned out to have the same polarity and to be quite similar in magnitude, whether or not self-citations were substracted.)

**Table 4:** The (Exp(ß)-1) values for logistic regressions.

Model	M1 (zero/lo)	M2 (lo/med-lo)	M3 (lo/med-hi)	M4 (lo/hi)
Dependent Var.	0 citesvs1–4 cites (lo)	1–4 cites (lo) vs5–9 cites (med-lo)	1–4 cites (lo)vs10–19 cites (med-hi)	1–4 cites (lo)vs20+ cites (hi)
Age	**0.494**	**0.490**	**0.786**	**1.439**
JIF	**1.229**	**0.514**	**0.776**	**1.114**
Auth_N	**0.007**	**0.002**	*0.002*	**−0.001**
Ref_N	**0.020**	**0.016**	**0.020**	**0.019**
Page_N	**−0.007**	**−0.014**	**−0.008**	
Sci	**0.249**	**0.475**	**0.887**	**2.050**
Review	**−0.373**	**−0.223**	*−0.008*	**0.914**
USA		**0.415**	**0.406**	**0.860**
OA	*−0.043*	**0.323**	**0.392**	**7.953**
Age*OA	**0.209**			*−0.032*
M		**0.889**	**0.716**	
CERN	**−0.211**			**1.306**
South				
Minho				
Queens	**0.476**			

**Figure 5:**
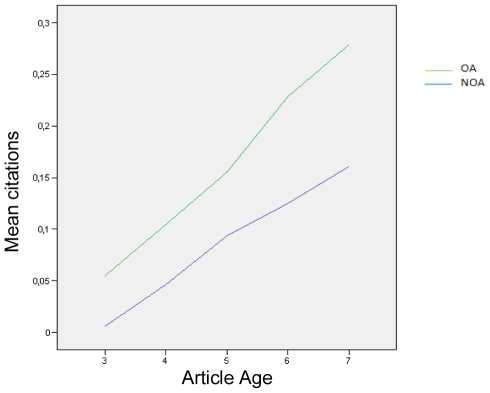
Interaction between OA and article age. Over and above the sum of the independent positive effects on citations of OA alone and of age alone, the size of this OA Advantage increases as articles get older. The interaction is illustrated here for the lo/hi (1–4/20+) citation range comparison (model M4) for articles that were from 3 years old (2006) to 7 years old (2002). (The comparison was made in 2009.)

14 predictor variables (plus one interaction variable) were used to predict the target variable (citation counts): (1) article age (Age), (2) journal impact factor (JIF), (3) number of co-authors (Auth_N), (4) number of references cited (Ref_N) , (5) number of pages (Page_N), (6) Science vs. Social Science (Sci), (7) Review article vs. ordinary article (Review), (8) US co-author vs. no US co-author (USA), (9) open access vs not (OA), (10) non-additive interaction between OA and Age (Age*OA), (11) OA mandated vs. not (M), (12) mandating institution CERN (CERN), (13) mandating institution Southampton ECS (South), (14) mandating institution U. Minho (Minho), (15) mandating institution Queensland U. Technology (Queens). Four logistic regression models estimated the size of the independent contribution of each of the 15 predictor variables to predicting the citation counts using four different cut-off values and comparison ranges (selected on the basis of the overall citation count distribution in **Figure 3**): (M1) articles with 0 vs lo (1–4) citations; (M2) lo (1–4) vs. med-lo (5–10) citations; (M3) lo (1–4) vs. med-hi (10–19) citations; (M4) lo (1–4) vs. hi (20+) citations. Note that OA is a significant independent contributor to citations in all but the lowest of these four citation ranges. The effect is displayed as a bar graph in **Figure 5**. (**Boldface values for** ***Exp(ß)*** **indicate differences significant at p<0.01** and *italic values indicate differences significant at 0.01≤p<0.05*.)

**Figure 4** shows that citations are, as is already well-known, positively correlated with the first eight variables listed earlier (Age, Journal Impact Factor, Authors, References, Pages, Science, Review, USA) – as well as with OA. Articles that are made OA have significantly higher citation counts. In this analysis the significant OA advantage is independent of the other variable; it is present in every citation range but highest in the highest citation range (1–4 citations vs 20+ citations): In other words, the OA advantage is strongest for highly cited articles. (The classification as ‘Review’ is derived from the Thomson-Reuters database, which uses number of references cited as its main criterion for classifying an article as a Review. As the number of references cited is another one of our predictor variables, there was probably some confounding of these two non-independent factors in our analysis. Citations came out as negatively correlated with the Review variable for the low-medium citation ranges in our analysis, so it was eliminated in further analyses.)

In our sample, articles by authors at the mandated institutions have higher than average citation counts; this effect is present only in the medium-high citation ranges (and is of course also influenced by the level of author compliance with the institutional Mandate, discussed further below). CERN articles have higher citation counts in the lowest and especially the highest citation range. However, when all CERN articles are excluded from our sample, there is no significant change in the other variables.

There is a significant interaction between Age and OA (Age*OA) for the lowest citation range comparison, zero/lo (0 vs. 1–4 citations) as well for the highest comparison, lo/hi (1–4 citations vs. 20 citations and more). Both the linear main effect of age and OA, and this nonlinear interaction are statistically significant. **Figure 5** illustrates the Age*OA interaction effect for the lo/hi range comparison using the means for OA and Non-OA citation counts for each article age. The pattern again confirms the OA advantage but also shows that in the lo/hi comparison range the advantage increases more for older articles, over and above what would be expected from age alone.

### Logistic regression by Impact Factor interval

**Figure 6:**
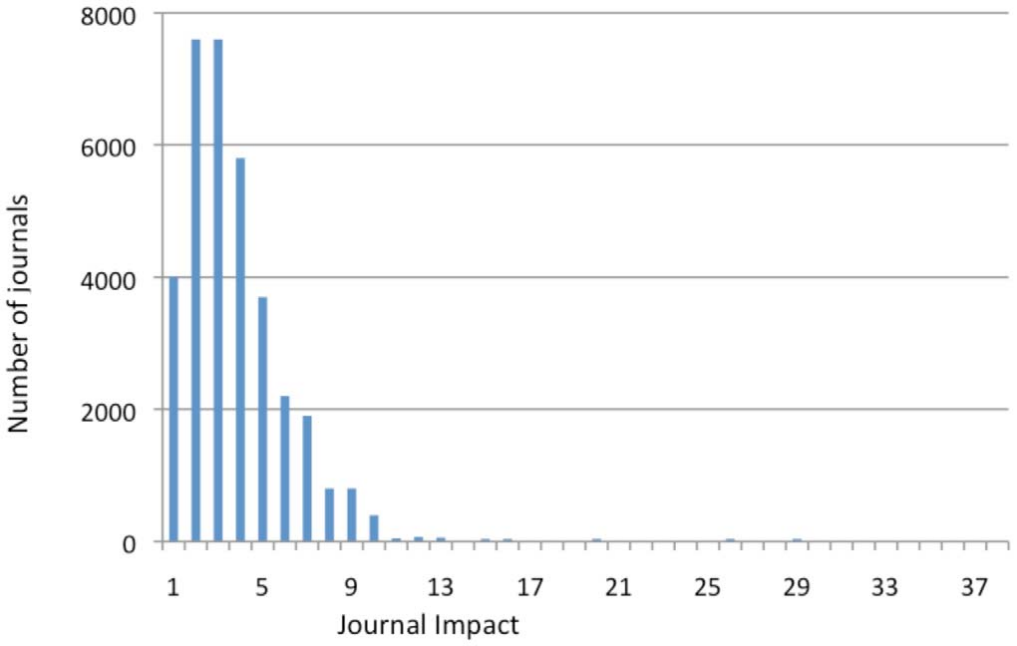
Distribution of Journal Impact Factors by Journal. As with the distribution of individual article citation counts (**Figure 3**), the distribution of journal impact factors (average citation counts) is highly skewed. Most journal JIFs fall between 0 and 5, with the peak between 2 and 3, followed by a long rapidly shrinking tail, tail with very few journals having a JIF greater than 10.

**Figure 7:**
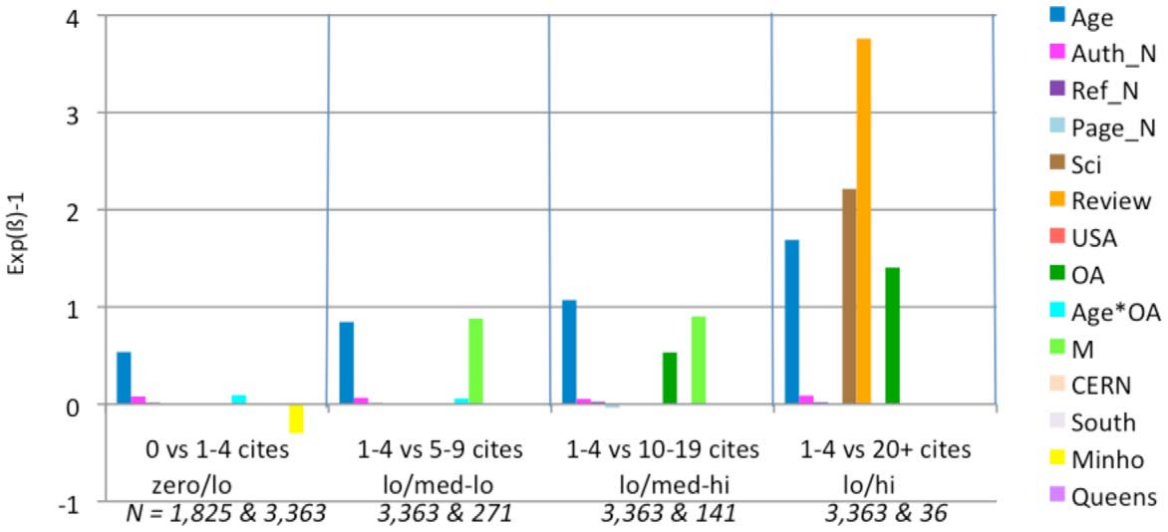
Exp(ß)-1 values for logistic regressions (Lowest JIF Range: 0.0–.0.63). (See **Figure 5** for explanation of analysis and interpretation.) In this lowest range of journal impact factors, the biggest factor contributing to citation in all citation range comparisons is article age. OA is an important contributor in the two upper range comparisons.

In order to compare articles published in comparable journals and to see the profile for journals in increasing impact ranges (see distribution, **Figure 6**), we divided our sample into 4 quartiles in terms of Journal Impact Factor (JIF), each range covering 25% of the articles:
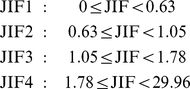
Only the top quartile contains journals with JIFs from 1.78 to 29.96. As we are also interested in the variability within this top quartile, we further subdivided it into two octiles, each covering 12.5% of the articles. (Subdividing more minutely would make the sample sizes too small to detect effects of interest.) This yielded a total of five ranges for the JIF variable:
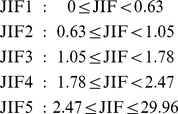
The same regression is done separately for each JIF range by controlling all the variables (except JIF). **Figures 7** **,** **8** **,** **9** **,** **10** **,** **11** (and **Appendix S2**: **Table S2a–S2e**) summarize the values of **Exp(ß)-1** corresponding to the controlled variables for each JIF range. (As noted earlier, our Exp(ß) values for these variables exhibit the same polarity and pattern whether or not we exclude self-citations from the citation count.)

**Figure 8 pone-0013636-g008:**
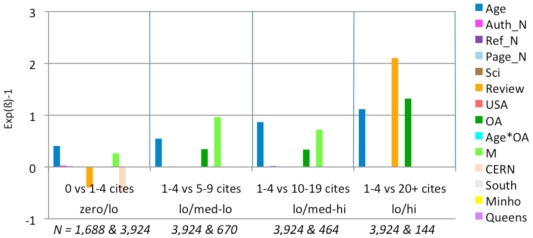
Exp(ß)-1 values for logistic regressions (JIF range 0.63–1.05). (See **Figure 5** for explanation of analysis and interpretation.) In the second lowest JIF range, article age continues to be the main factor in all four citation ranges, with OA emerging and growing in the top three.

**Figure 9 pone-0013636-g009:**
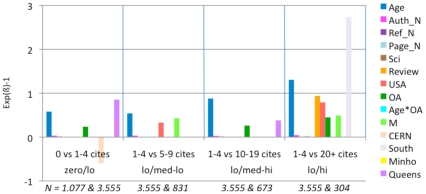
Exp(ß)-1 values for logistic regressions (JIF range 1.05–1.78). (See **Figure 5** for explanation of analysis and interpretation.) In this middle range of journal JIFs, article age continues to be influential, and OA is a significant factor in three of the four citation ranges.

**Figure 10 pone-0013636-g010:**
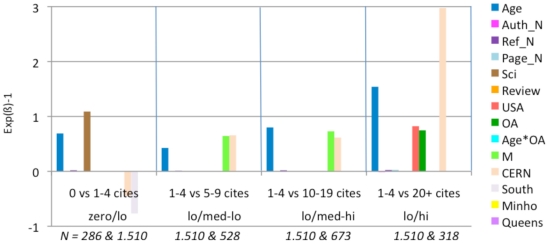
Exp(ß)-1 values for logistic regressions (JIF range 1.78–2.47). (See **Figure 5** for explanation of analysis and interpretation.) In this next-to-highest JIF range, OA has its effect only in the top range (lo/hi).

**Figure 11:**
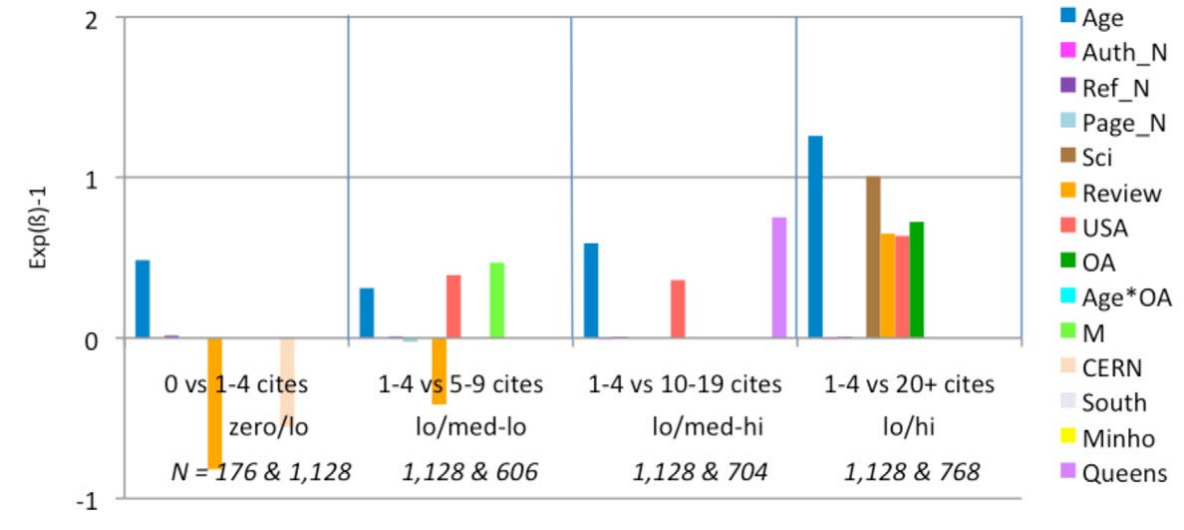
Exp(ß)-1 values for logistic regressions (JIF 2.47–29.96). (See **Figure 5** for explanation of analysis and interpretation.) In this, the highest JIF range, article age again increases citations in all ranges, whereas OA again has its effect only in the top range (lo/hi) (Note the anomalous effect of the “Review” variable; this is probably because it is confounded with the Reference count variable; when Review was removed in further analyses, the pattern of the other variables, and in particular OA, was unchanged.)

When articles are published in a low JIF journal, citation counts for their individual articles are positively correlated with Age, References, Authors, OA and M. The OA advantage is greater in the higher citation ranges. For the lowest range of individual article citations, the Age*OA interaction is significant, but OA itself is not.

For articles in journals with JIFs between 0.63 and 1.05, the pattern is quite similar, except that the Age*OA interaction is absent and OA itself (alongside Age, as separate variables) is significant.

For articles in journals with JIFs between 1.05 and 1.78, the pattern is again quite similar. The USA and Review variables now also correlate with citation increase.

For journals with JIFs between 1.78 and 2.47, longer articles (more pages) have more citations. Here the OA advantage is significant only in the highest citation count ranges. The number of authors is also less correlated with increased citations as the citation range gets higher. CERN and QUT have a citation advantage in this JIF range. However, removing the articles from these institutions does not alter the pattern for the other variables.

For journals with JIFs between 2.47 and 29.96. The OA advantage is again significant for the highest citation ranges. (The increased citations for USA and Review articles also increase in significance). In this JIF range, CERN has a citation advantage in medium-high citations ranges. Removing the articles from this institution, however, does not change the pattern for the other variables.

Overall, OA is correlated with a significant citation advantage for all journal JIF intervals as well as for the sample as a whole. This advantage is greatest for the highest citation ranges. When regressions are done separately for the different JIF ranges, the Age*OA interaction disappears, but OA and Age (as separate variables) remain significant. (There is no significant effect of a specific institution compared to the rest of the institutions, hence there is no need to exclude any specific institution from our sample.)

## Discussion

This study confirms that the OA advantage is a statistically significant, independent positive increase in citations, even when we control the independent contributions of many other salient variables (article age, journal impact factor, number of authors, number of pages, number of references cited, Review, Science, USA author). All these other variables are of course correlated with citation counts, so the fact that OA continues to correlate significantly with an independent positive increase in citation counts even when the contributions of all these other correlates are calculated independently means that the OA Advantage is not just a bias arising from either a random or a systematic imbalance in the other correlates of citations.

Moreover, the OA advantage is just as great when the OA is mandated (with mandate compliance rate ∼60%) as when it is self-selective (self-selection rate ∼15%). This makes it highly unlikely that the OA advantage is either entirely or mostly the result of an author bias toward selectively self-archiving higher quality – hence higher citability – articles. Nor are the main effects the result of institutional citation advantages, as the institutions were among the independent predictor variables in the logistic regression; the outcome pattern and significance is also unaltered by removing CERN, the only one of the four institutions that might conceivably have biased the outcome because its papers were all in one field and tended to be of higher quality, hence higher citability overall.

Since, with the exception of our one unidisciplinary institute – CERN (high energy physics) – the pluridisciplinary articles from the three other mandated institutional repositories are mostly not in fields that habitually self-archive their unrefereed preprints well before publication (as many in high energy physics do), nor in fields that already have effective OA for their published postprints (as astronomy does: [9], ), it is also unlikely that the OA advantage is either entirely or mostly just an early-access (prepublication) advantage [33], [34]. This will eventually be testable once there are enough reliable data available on deposit-date, relative to publication-date, for a large enough body of self-archived OA articles. In any case, an early-access advantage in a preprint self-archiving field translates into a generic postpublication OA advantage in that vast majority of fields in which authors do not self-archive their prepublication preprints and so their published postprints are accessible only to subscribers – except if they have also been self-archived. The OA mandates all apply only to refereed postprints, self-archived upon publication, not to pre-refereeing preprints, self-archived before publication.

This study confirms that the OA advantage is substantially greater for articles that have successfully met the quality standards of higher-impact journals and it is also greater in the higher-citation ranges for individual papers within each journal-impact level. The typical Pareto distribution for citations whereby the top 10–20% of articles receive about 80–90% of all citations [35], is present in our own sample of 708,219 articles extracted from Thomson-Reuters from 1998 to 2007: about 20% of *articles* received about 80% of all citations. In addition, 10% of *journals* receive 90% of all citations.

The implication is that OA itself will not make an unusable (hence uncitable) paper more used and cited (although the proportion of uncited papers has been diminishing with time; [31]). But wherever there are subscription-based constraints on accessibility, providing OA will increase the usage and citation of the more usable and citable papers, probably in proportion to their importance and quality, hence citability. We accordingly infer from our results that the most likely cause of the OA citation advantage is not *author self-selection* toward making more citable articles OA, but *user self-selection* toward using and citing the more citable articles – once OA self-archiving has made them accessible to all users, rather than just to those whose institutions could afford subscription access. In other words, we conclude that the OA advantage is a *quality advantage*, rather than a *quality bias*: it is not that the higher quality articles – the ones that are more likely to be selectively cited anyway – are more likely to be made OA self-selectively by their authors, but that the higher quality articles that are more likely to be selectively cited are made more accessible, hence more citable, by being made OA.

Our results also suggest the possibility that mandated OA might have some further independent citation advantage of its own, over self-selected OA – but until and unless this effect is replicated, it is more likely that this small, previously unreported effect was due to chance or sampling error. If there does indeed prove to be an independent “mandate advantage” over and above OA itself, a possible interpretation would be the reverse of the self-selection hypothesis: There may be a higher proportion of higher-quality work among the 80% that are not being made OA on a self-selective basis today than among the 20% that are; the result is that the OA mandates serve to help bring this “cream of science” to the top.

It also needs to be noted that some of the factors contributing to the OA advantage are permanent, whereas others will shrink as OA rises from its current 15–20% level and will disappear completely at 100% OA. All *competitive advantage* of OA over non-OA (because OA is more accessible) will of course vanish at 100% OA (as will the possibility of concurrent measurement of the OA Advantage). Any *self-selective bias* (whether positive or negative) will likewise disappear at 100% OA. What will remain will be the *quality advantage* itself (the tendency of researchers to selectively use and cite the best research, if they can access it), but maximized by leveling the playing field, making everything accessible to every user online.

There will continue to be the early-access advantage in fast turnaround fields: It is not that making findings accessible earlier merely gets them their citation “quota” earlier; providing OA earlier significantly increases that quota, probably by both accelerating and broadening the uptake of the findings in further research [33]. And even after the competitive advantage is gone because all articles are OA, the *download advantage* will continue to be enjoyed by all articles [36], [37] (thereby potentially influencing research even where it does not generate citations), while the quality advantage will see to it that for the best work, increased downloads are translated into uptake, usage and eventual increased citations. (Higher download counts earlier on have been found to be correlated with, hence predictive of, increased citation counts later; [38].)

### Summary and Conclusion

The assumption that increasing access to research will increase its usage and impact is the main rationale for the worldwide OA movement. Many prior studies have by now shown across all fields that journal articles that are made freely accessible to all potential users are cited significantly more than articles that are accessible only to subscribers. There is prior evidence for a self-selection bias toward the preferential self-archiving of higher quality articles in a few special fields (such as astronomy and some areas of physics) where most articles are made OA in unrefereed preprint form long before they are refereed and published, and where the published version is effectively accessible to all potential users as soon as it is published. Authors may indeed be more reluctant to make the preprints of papers about which they have doubts freely accessible online before they are refereed [29], [33]. But we have now shown that for most other fields (i) the OA Advantage remains just as high for mandatory self-archiving as for self-selected self-archiving and that (ii) this is not an artifact of systematic biases in other correlates of citation counts. Both the self-archiving and the mandates apply to refereed postprints, upon acceptance for publication, not to unrefereed preprints.

Hence the OA Advantage is real, independent and causal. It is indeed true that the size of the advantage is correlated with quality, just as citations themselves are correlated with quality (the top 20% of articles receiving about 80% of all citations); but we infer that the real cause of the higher OA advantage for the more citable articles is not a quality bias from author self-selection but the quality advantage of the more citable articles, an advantage that OA *enhances* by maximizing accessibility, and thereby also citability. On a playing field leveled by OA, users can selectively access, use and cite those articles that they judge to be of the highest relevance and quality, no longer constrained by their accessibility.

Overall, only about 15–20% of articles are being spontaneously self-archived today, self-selectively. To reach 100% OA globally, researchers' institutions and funders need to mandate self-archiving, as they are now increasingly beginning to do. We hope that this demonstration that the OA Impact Advantage is real and causal will provide further incentive and impetus for the adoption of OA mandates worldwide in order to ensure that research can at last achieve its full impact potential, no longer constrained by today's needless limits on its accessibility to its intended users [39]–[42].

To measure that maximized research impact, we and others are already developing new OA metrics for monitoring, analyzing, evaluating, crediting and rewarding research productivity and progress [18], [36], [38], [43]–[52]. Hence there is no need to have any penalties or sanctions for non-compliance with OA self-archiving mandates. As the experience of Southampton ECS, Minho, QUT and CERN has already demonstrated, OA mandates, together with OA's own intrinsic rewards (enhanced research access, usage and impact), will be enough to reinforce the causal connection between providing access and reaping its impact, through the research community's existing system for evaluating and rewarding research productivity. In the online era, researchers' own “mandate” will no longer just be “publish-or-perish” but “self-archive to flourish.”

## Supporting Information

Appendix S1 OA Impact Advantage for each Institution. Figure 2 showed the mean log citation ratios for O/Ø, OM/OS, OS/ØS, OM/ØM, OM/Ø, OS/Ø and OM/OS for the four institutions together. The outcome was that the Open Access (OA) citation advantage was present and roughly equal whether the OA was Self-Selective (S) or Mandated (M). That showed that the OA Advantage is not merely an artifact of author self-selection. This appendix shows the results for each institution separately. As will be evident, the pattern for the individual institutional data is largely the same as it is for the average across the four institutions.
(0.21 MB DOC)

Appendix S2 Multiple regression by JIF - Beta values. The multiple logistic regression we applied to our total sample of journals is applied here separately to the journals in each JIF (Journal Impact Factor) range by including all the other 14 predictor variables, apart from JIF itself. Tables S2a–S2e summarize the values of Exp(β)-1 corresponding to the predictor variables for each JIF range. The results were discussed in Figures 7–11. In sum, they show that whereas citation counts grow with an article's age across all the citation range comparisons for our four models (zero/low, low/medium1, low/medium2, low/high), OA's contribution tends to be more on the high-citation end, being greater in the higher JIF range (JIF4–JIF5) among journals and in the low/high range comparisons (M4) among articles.
(0.13 MB DOC)
