# Supplementary material for: Self-Selected or Mandated, Open Access Increases Citation Impact for Higher Quality Research
Source: PLoS One. 2010 Oct 18;5(10):e13636. doi: 10.1371/journal.pone.0013636 (PMC2956678; doi:10.1371/journal.pone.0013636)
Supplement: Appendix S1 — OA Impact Advantage for each Institution. Figure 2 showed the mean log citation ratios for O/Ø, OM/OS, OS/ØS, OM/ØM, OM/Ø, OS/Ø and OM/OS for the four institutions together. The outcome was that the Open Access (OA) citation advantage was present and roughly equal whether the OA was Self-Selective (S) or Mandated (M). That showed that the OA Advantage is not merely an artifact of author self-selection. This appendix shows the results for each institution separately. As will be evident, the pattern for the individual institutional data is largely the same as it is for the average across the four institutions. (0.21 MB DOC) [file pone.0013636.s001.doc]

## Appendix S1. OA Impact Advantage for each Institution

**Figure 2** showed the mean log citation ratios for O/Ø, OM/OS, OS/ØS, OM/ØM, OM/Ø, OS/Ø and OM/OS for the four institutions together. The outcome was that the Open Access (OA) citation advantage was present and roughly equal whether the OA was Self-Selective (S) or Mandated (M). That showed that the OA Advantage is not merely an artifact of author self-selection. This appendix shows the results for each institution separately. As will be evident, the pattern for the individual institutional data is largely the same as it is for the average across the four institutions.


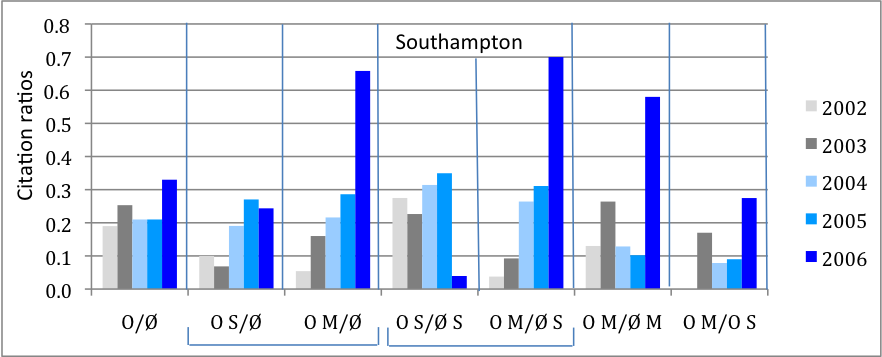


**Figure S1a. OA Impact Advantage for Self-Selected vs Mandatory OA for Southampton ECS**. (This figure is similar to **Figure 2**).


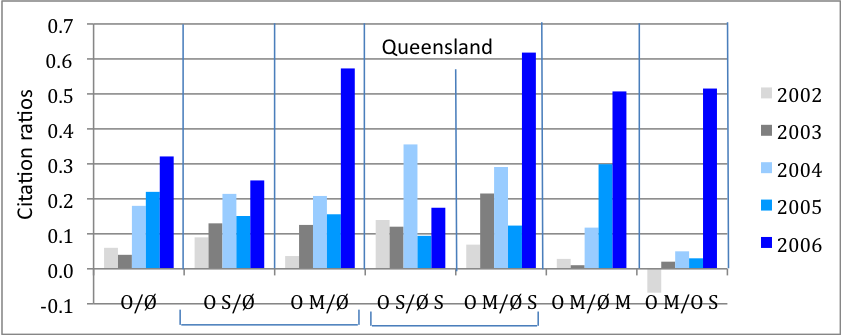


**Figure S1b. OA Impact Advantage for Self-Selected vs Mandatory OA for Queensland University of Technology (QUT).** (This figure is similar to **Figure 2**).

***
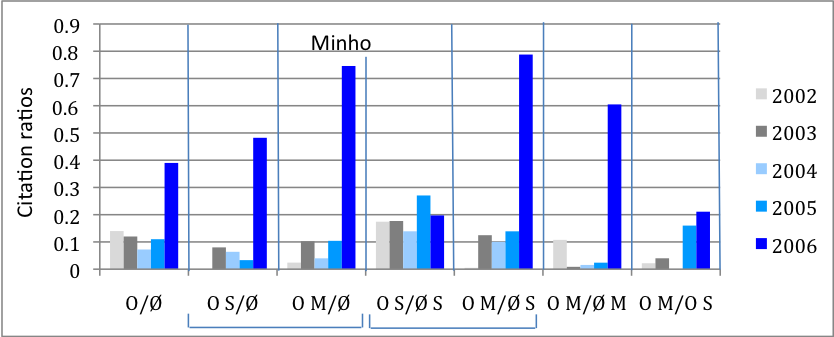
***

**Figure S1c. OA Impact Advantage for Self-Selected vs Mandatory OA for Minho.** (This figure is similar to **Figure 2**).


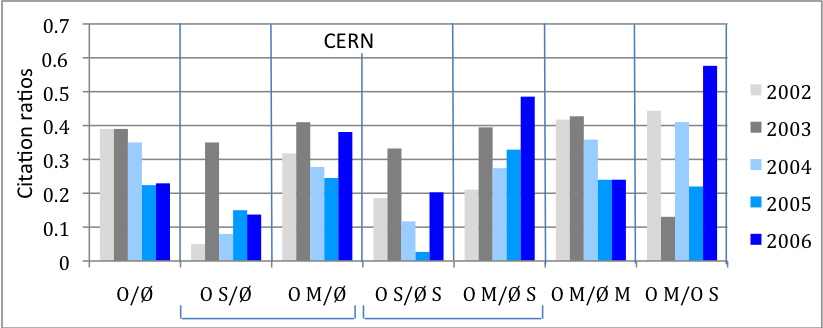


**Figure S1d. OA Impact Advantage for Self-Selected vs Mandatory OA for CERN.** (This figure is similar to **Figure 2**).
