# Supplementary material for: Self-Selected or Mandated, Open Access Increases Citation Impact for Higher Quality Research
Source: PLoS One. 2010 Oct 18;5(10):e13636. doi: 10.1371/journal.pone.0013636 (PMC2956678; doi:10.1371/journal.pone.0013636)
Supplement: Appendix S2 — Multiple regression by JIF - Beta values. The multiple logistic regression we applied to our total sample of journals is applied here separately to the journals in each JIF (Journal Impact Factor) range by including all the other 14 predictor variables, apart from JIF itself. Tables S2a–S2e summarize the values of Exp(β)-1 corresponding to the predictor variables for each JIF range. The results were discussed in Figures 7– 11. In sum, they show that whereas citation counts grow with an article's age across all the citation range comparisons for our four models (zero/low, low/medium1, low/medium2, low/high), OA's contribution tends to be more on the high-citation end, being greater in the higher JIF range (JIF4–JIF5) among journals and in the low/high range comparisons (M4) among articles. (0.13 MB DOC) [file pone.0013636.s002.doc]

## Appendix S2. Multiple regression by JIF – Beta values

The multiple logistic regression we applied to our total sample of journals is applied here separately to the journals in each JIF (Journal Impact Factor) range by including all the other 14 predictor variables, apart from JIF itself. **Tables S2a-S2e** summarize the values of **Exp(ß)-1** corresponding to the predictor variables for each JIF range. The results were discussed in Figures 7-11. In sum, they show that whereas citation counts grow with an article’s age across all the citation range comparisons for our four models (zero/low, low/medium1, low/medium2, low/high), OA’s contribution tends to be more on the high-citation end, being greater in the higher JIF range (JIF4-JIF5) among journals and in the low/high range comparisons (M4) among articles.

1. JIF1 (JIF < 0.63)

| Model | M1 (zero/lo) | M2 (lo/med-lo) | M3 (lo/med-hi) | M4 (lo/hi) |
| --- | --- | --- | --- | --- |
| Dependent Var. | 0 cites  vs  1-4 cites (lo) | 1-4 cites (lo) vs  5-9 cites (med-lo) | 1-4 cites (lo)  vs  10-19 cites (med-hi) | 1-4 cites (lo)  vs  20+ cites (hi) |
| Age | **0.537** | **0.847** | **1.071** | **1.689** |
| Auth_N | **0.079** | **0.066** | *0.054* | **0.087** |
| Ref_N | **0.017** | **0.013** | **0.026** | **0.020** |
| Page_N |  |  | **-0.038** |  |
| Sci |  |  |  | *2.214* |
| Review |  |  |  | *3.760* |
| USA |  |  |  |  |
| OA |  |  | *0.533* | *1.406* |
| Age*OA | **0.093** | *0.059* |  |  |
| M |  | **0.881** | **0.902** |  |
| CERN |  |  |  |  |
| South |  |  |  |  |
| Minho | **-0.299** |  |  |  |
| Queens |  |  |  |  |

**Table S2a. Exp(ß)-1 values for logistic regressions for JIF1**

1. JIF2 (0.63 <= JIF < 1.05)

| Model | M1 (zero/lo) | M2 (lo/med-lo) | M3 (lo/med-hi) | M4 (lo/hi) |
| --- | --- | --- | --- | --- |
| Dependent Var. | 0 cites  vs  1-4 cites (lo) | 1-4 cites (lo) vs  5-9 cites (med-lo) | 1-4 cites (lo)  vs  10-19 cites (med-hi) | 1-4 cites (lo)  vs  20+ cites (hi) |
| Age | **0.407** | **0.548** | **0.869** | **1.117** |
| Auth_N | **0.028** |  | **0.007** |  |
| Ref_N | **0.016** | **0.012** | **0.018** | **0.011** |
| Page_N |  |  |  |  |
| Sci |  |  |  |  |
| Review | *-0.395* |  |  | **2.106** |
| USA |  |  |  |  |
| OA |  | **0.346** | *0.337* | **1.322** |
| Age*OA |  |  |  |  |
| M | *0.265* | **0.963** | **0.722** |  |
| CERN | **-0.489** |  |  |  |
| South |  |  |  |  |
| Minho |  |  |  |  |
| Queens |  |  |  |  |

**Table S2b. Exp(ß)-1 values for logistic regressions for JIF2**

1. JIF3 (1.05 <= JIF < 1.74)

| Model | M1 (zero/lo) | M2 (lo/med-lo) | M3 (lo/med-hi) | M4 (lo/hi) |
| --- | --- | --- | --- | --- |
| Dependent Var. | 0 cites  vs  1-4 cites (lo) | 1-4 cites (lo) vs  5-9 cites (med-lo) | 1-4 cites (lo)  vs  10-19 cites (med-hi) | 1-4 cites (lo)  vs  20+ cites (hi) |
| Age | **0.581** | **0.540** | **0.879** | **1.305** |
| Auth_N | **0.032** | **0.033** | *0.026* | **0.041** |
| Ref_N | **0.012** | **0.007** | **0.013** | **0.009** |
| Page_N |  |  |  | **0.026** |
| Sci |  |  |  |  |
| Review |  |  |  | *0.939* |
| USA |  | **0.330** |  | **0.791** |
| OA | *0.236* |  | *0.263* | ***0.449*** |
| Age*OA |  |  |  |  |
| M |  | **0.428** |  | *0.492* |
| CERN | **-0.599** |  |  |  |
| South |  |  |  | **2.734** |
| Minho |  |  |  |  |
| Queens | **0.856** |  | *0.382* |  |

**Table S2c*.* Exp(ß)-1 values for logistic regressions for JIF3**

1. JIF4 (1.74 <= JIF < 2.47)

| Model | M1 (zero/lo) | M2 (lo/med-lo) | M3 (lo/med-hi) | M4 (lo/hi) |
| --- | --- | --- | --- | --- |
| Dependent Var. | 0 cites  vs  1-4 cites (lo) | 1-4 cites (lo) vs  5-9 cites (med-lo) | 1-4 cites (lo)  vs  10-19 cites (med-hi) | 1-4 cites (lo)  vs  20+ cites (hi) |
| Age | **0.690** | **0.427** | **0.800** | **1.540** |
| Auth_N |  |  |  | **-0.006** |
| Ref_N | **0.020** | **0.010** | **0.019** | **0.024** |
| Page_N |  |  |  | *0.028* |
| Sci | *1.090* |  |  |  |
| Review |  |  |  |  |
| USA |  |  |  | **0.822** |
| OA |  |  |  | **0.747** |
| Age*OA |  |  |  |  |
| M |  | **0.645** | **0.729** |  |
| CERN | *-0.446* | *0.657* | **0.615** | **2.974** |
| South | *-0.767* |  |  |  |
| Minho |  |  |  |  |
| Queens |  |  |  |  |

**Table S2d. Exp(ß)-1 values for logistic regressions for JIF4**

1. JIF5 (2.47 <= JIF < 29.96)

| Model | M1 (zero/lo) | M2 (lo/med-lo) | M3 (lo/med-hi) | M4 (lo/hi) |
| --- | --- | --- | --- | --- |
| Dependent Var. | 0 cites  vs  1-4 cites (lo) | 1-4 cites (lo) vs  5-9 cites (med-lo) | 1-4 cites (lo)  vs  10-19 cites (med-hi) | 1-4 cites (lo)  vs  20+ cites (hi) |
| Age | **0.484** | **0.312** | **0.590** | **1.259** |
| Auth_N |  |  | *-0.002* | **-0.005** |
| Ref_N | **0.016** | **0.010** | **0.007** | **0.009** |
| Page_N |  | **-0.024** |  |  |
| Sci |  |  |  | *1.007* |
| Review | **-0.818** | *-0.414* |  | *0.650* |
| USA |  | **0.391** | **0.360** | **0.635** |
| OA |  |  |  | **0.722** |
| Age*OA |  |  |  |  |
| M |  | **0.468** |  |  |
| CERN | **-0.554** |  |  |  |
| South |  |  |  |  |
| Minho |  |  |  |  |
| Queens |  |  | **0.751** |  |

**Table S2e.  Exp(ß)-1 values for logistic regressions for JIF5**
